# Mechanisms of alignment: shared control, social cognition and metacognition

**DOI:** 10.1098/rstb.2021.0362

**Published:** 2023-02-13

**Authors:** Greta Gandolfi, Martin J. Pickering, Simon Garrod

**Affiliations:** ^1^ Department of Psychology, The University of Edinburgh, 7 George Square, Edinburgh EH8 9JZ, UK; ^2^ Department of Neuroscience and Psychology, University of Glasgow, Glasgow G12 9YR, UK

**Keywords:** dialogue, alignment, abstract concepts, shared control, metacognition, social cognition

## Abstract

In dialogue, speakers process a great deal of information, take and give the floor to each other, and plan and adjust their contributions on the fly. Despite the level of coordination and control that it requires, dialogue is the easiest way speakers possess to come to similar conceptualizations of the world. In this paper, we show how speakers align with each other by mutually controlling the flow of the dialogue and constantly monitoring their own and their interlocutors' way of representing information. Through examples of conversation, we introduce the notions of shared control, meta-representations of alignment and commentaries on alignment, and show how they support mutual understanding and the collaborative creation of abstract concepts. Indeed, whereas speakers can share similar representations of concrete concepts just by mutually attending to a tangible referent or by recalling it, they are likely to need more negotiation and mutual monitoring to build similar representations of abstract concepts.

This article is part of the theme issue ‘Concepts in interaction: social engagement and inner experiences’.

## 1. Introduction

Dialogues are everyday cooperative joint activities, involving more than one person who seeks to comprehend each other. Given how common it is and how natural it feels to take part in dialogues (from ordering a coffee in a bar to chatting with friends over a meal), one might risk overlooking the cognitive challenges that dialogue offers to speakers. Speakers must figure out what to say, when to say it and what to do when the conversation goes off track. During unstructured conversations, people discuss different and unrelated topics, jumping from one to another, and continually taking and giving the floor to their interlocutor. Nevertheless, in most cases, speakers seem to know what is appropriate to say, given the context and the people involved, even when they cannot plan it in advance. More formally, dialogue requires opportunistic planning (the ability to adapt to new information), turn-taking (the ability to give the right contribution at the right time), and audience design (the ability to shape contributions to the needs of different interlocutors). However, for unimpaired speakers who have mastered a common language, talking to each other is—most of the time—straightforward [1]. But the phenomenological easiness of dialogue contrasts with the amount of control it requires to run smoothly. In fact, shared control allows speakers to guide the conversation toward mutual understanding.

Speakers reach mutual understanding when their understanding is aligned—that is, when they focus on the same aspects of the world and conceptualize them in the same way. Interlocutors do not reach alignment in isolation, but through interaction, by manipulating each other's contributions, in a collaborative fashion. We propose an extension of the interactive alignment model [1,2], specifically in relation to Pickering and Garrod's book [3]. In particular, we argue that interlocutors share control over the dialogue by continuously monitoring and comparing their own and their interlocutor's contributions, by drawing on metacognition and social cognition, and specifically by meta-representing whether they believe they and their interlocutor are aligned or not. This process allows speakers to build similar representations and align on their way of understanding complex phenomena in the world without having to build elaborate inferential systems of beliefs about their own and their interlocutor's understanding.

We also argue that dialogue is particularly crucial for aligning on abstract concepts as compared to concrete ones. Indeed, aligning on concrete concepts might not even need any linguistic exchange—it may be enough for a lamp to be visible to both interlocutors for them to align on the concept LAMP. But aligning on abstract concepts, such as FREEDOM, usually requires speakers to compare their own perspectives and negotiate a new one. Because of their contextual, cultural and individual variability [4], abstract concepts require speakers to agree on their representations and they do so by expressing metacognitive states about their own representations, about their interlocutor's representations, and about the degree of alignment between their own and their interlocutor's representations.

## 2. Speakers align

In dialogue, speakers deal with different types of information. First, they interpret language with respect to a model of the situation under discussion (using what we call a situation model). Second, they need to keep track of the structure of the dialogue—for example, whose turn it is to talk (using what we call a dialogue game model). Finally, they process linguistic information that they need to comprehend and produce utterances. We argue that successful dialogue involves the alignment of these different types of information. However, for the purposes of the paper, we mainly focus on the relationship between linguistic and situation model alignment.

Consider the following extract [5]. (As in all examples, we kept the original annotations and used female or male pronouns arbitrarily, unless the original source makes the gender clear. Moreover, we used the original identifications of the speakers.)^1^

1.
A: Oh, I have the- I have one class in the e:vening.
B: On Mondays?
A: Y-uh::: Wednesdays.=
B: =Uh-Wednesday,=
A: =En it's like a Mickey Mouse course.

Let us start with an informal analysis. A and B were talking about their schedule. B seemed to know that A was busy on some evenings, but, when A said that she had ‘one class’, B was unsure about which evening A was referring to. Given some previous knowledge about A's schedule, B asked ‘On Mondays?’. After A's correction ‘Y-uh::: Wednesdays.=’, A and B realised that Wednesday evening was not a good time to do what they were planning together, and A continued the conversation.

Now let us analyse the extract in terms of alignment, which is a property of the relationship between representations. Two speakers are aligned when they share similar mental representations of what they are discussing (situation model alignment) and the language they are using (linguistic alignment). Linguistic and situation model alignment are highly interconnected. Research on alignment indicates that speaking in the same way (using the same linguistic representations) leads people to think in the same way (representing the world in the same way). For example, Garrod & Anderson [6] showed how people converge on the same description scheme for communicating their positions in a maze (e.g. matrix or line scheme) by repeating their interlocutors' linguistic choices. This means that the speakers converge on the same conceptualization of the maze (situation model alignment), by aligning at a linguistic level (linguistic alignment). Alignment, in fact, can percolate through different levels of representation [1,3,7].

Consider linguistic representations first. When A corrected B, she activated the lexical entry *Wednesday* and uttered ‘Y-uh::: Wednesdays.=’*.* B also activated the same lexical entry and repeated the word. A and B were aligned on the linguistic representation of *Wednesday* (i.e. its phonology, syntax, and semantics) because A produced it, and B comprehended it. Indeed, linguistic alignment explains why speakers often repeat each other's lexical [8] and syntactic choices [9]. In other words, A and B's linguistic behaviour matched because their lexical representations were aligned^2^. In addition, A activated other concepts related to WEDNESDAY, and similar concepts were likely to be activated by B (assuming A and B shared cultural context). If, for example, A and B were members of a yoga society providing free classes on Wednesday evenings, then it would have been likely that A and B both activated the concept YOGA-CLASS, along with the concept WEDNESDAY. Therefore, A and B would not only have been aligned at a lexical level but also at the semantic level.

Now consider situation model representations. At the beginning of the conversation, A knew that she had a class on Wednesday, therefore she represented HAS-CLASS (A, WEDNESDAY). At the same time, B represented HAS-CLASS (A, MONDAY). After A's correction ‘Y-uh::: Wednesdays.=’, A and B both represented HAS-CLASS (A, WEDNESDAY), and so they were aligned with respect to that representation.

As we saw, linguistic alignment is often a consequence of automatic co-activation patterns and it can eventually lead to situation model alignment [6]. However, situation model alignment often depends also on shared control of the dialogue. Since the linguistic representations that A used to produce ‘Y-uh::: Wednesdays.=’ were the same as the ones B used to understand it, A's utterance primed B's representation (WEDNESDAY) and led to her utterance. Thus, B aligned with A on the representation of HAS-CLASS (A, WEDNESDAY) partly as a consequence of this linguistic alignment. But situation model alignment also partly occurred because B asked A to disambiguate her utterance ‘Oh, I have the- I have one class in the e:vening’, by offering a repair ‘On Mondays?’. In the following section, we explain the mechanisms of shared control that lead to situation model alignment.

## 3. Speakers control the dialogue together

**Figure 1.**
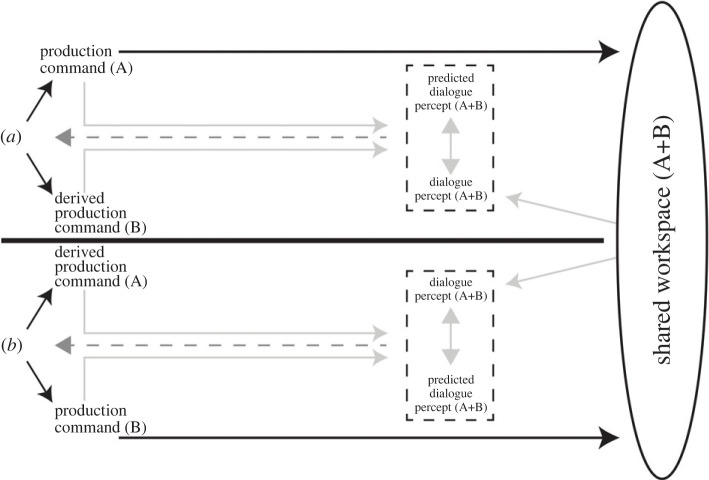
A model of the dialogue control system (simplified reproduction from Pickering & Garrod [3]). A's and B's production commands lead to utterance components (solid black lines) that feed into the dialogue recorded in the shared workspace. A's and B's comprehension of the dialogue (light grey left-facing arrows) is compared to what A and B predict (light grey right-facing arrows), through the derived production commands. The comparisons between dialogue percepts (what is perceived) and predicted dialogue percepts (what is predicted) can lead to changes in the A and B's utterance components and to changes in self-predictions as well as changes in other-predictions.

Speakers are able to act together on what Pickering and Garrod [3,12] called the shared workspace: the set of salient signs and associated context that are at both the speakers' disposal. Thus, the shared workspace includes speakers’ contributions as well as other elements that they both attend to, such as facial expressions and gestures, or objects that they are referring to. Speakers can add signs to it or can act on what is already there, by commenting, discussing and rephrasing it. We use a schema (figure 1) to illustrate how Example 1 works in these terms of shared control.

When A said ‘Oh, I have the- I have one class in the e:vening’, the utterance entered the shared workspace and became available to B. Each speaker planned his/her contributions, by generating a production command (roughly, the intention to communicate something) which was used to produce the actual utterance, via semantic, syntactic and phonological processing (black arrows in figure 1). In Example 1, A used the production command to articulate ‘Oh, I have the- I have one class in the e:vening’ by activating the relevant linguistic representations. In parallel, A and B generated a derived production command, which corresponded to what they expected to hear from each other and from themselves.

In this case, A predicted A's own utterance (though it is possible that a mismatch led to A reformulating her production command after ‘the-’). At the same time, B predicted what B expected to hear from A by putting himself in A's shoes. Predicting each other's contributions allows well-aligned speakers (in terms of linguistic and situation model representations) to finish the sentence of their interlocutor, or to suggest a specific word that they cannot recall [13].

Predictions (predicted dialogue percepts) were then compared to what was added to the shared workspace (dialogue percepts). B compared what A said and what B expected and registered whether there was a significant mismatch or not. Presumably, B predicted A would have specified which weekday the class was on. Therefore, B received a small prediction error, hence computing the production command to utter the clarification request ‘On Mondays?’. At the same time, A predicted B's utterance and noticed a greater mismatch (since her representation included WEDNESDAY, whereas B's representation did not). This mismatch led to A reformulating her plans and uttering ‘Y-uh::: Wednesdays.=’. After A's correction, as we have seen, A and B ended up sharing a similar representation of HAS-CLASS (A, WEDNESDAY). A may have predicted an indication of understanding from B, which B gave by repeating the ‘=Uh-Wednesday,=’. Notice that both A and B did not predict exactly what their interlocutor was going to say, but rather made more limited predictions (grey right-facing arrows in figure 1). We also cannot of course be certain how much is predicted and how much is determined after the relevant utterance, especially since we do not have precise information about timing.

Simulating others’ contributions is central to dialogue control and success. First, it means that the production system is kept activated during comprehension. B was not a passive listener, because, as A uttered her first contribution, B acted as if he were the producer. This allowed A and B to switch their roles. They of course switched roles repeatedly: they could each take the floor easily and were always prepared to contribute to the dialogue. Second, linguistic alignment makes predictions more straightforward. Since prediction requires speakers to simulate their interlocutor's production processes, having similar representations makes simulation simpler and more accurate, so that the subsequent process of alignment is more straightforward.

## 4. Speakers act on their own and their interlocutor's representations

We have seen how speakers control the dialogue to adapt their plans and utterances to each other's needs. What allows them to exercise this control is their ability to compare their representations and monitor whether they are aligned or not^3^. According to Pickering & Garrod [3], speakers keep track of alignment by meta-representing it. Such meta-representations drive their use of commentaries that help them to shape and guide following contributions. Our use of the term commentary is related to what other psycholinguistic and conversation analytic theories call ‘backchannels’ [14–17] or ‘repair initiators’ [18–21]. We refer to commentaries because our concern is with their function for the responder, which is to provide an indication that he or she meta-represents alignment (a positive commentary) or misalignment (a negative commentary) with the main speaker. By contrast, a ‘backchannel’ refers to a response occurring during a main contribution (and therefore excludes responses in which the responder ‘takes the floor’). A ‘repair initiator’ is an instance of a negative commentary that leads to the main contributor reformulating, and of course not all negative commentaries have this effect. The term commentary, therefore, allows us to consider a broad range of contributions and focus on the interlocutors' mental representations rather than behaviour.

Consider the following extract [22]:

2.
JIM: a=nd uh, you know, I think, I think that, one of the reasons that there's been no focus is because, (H) oh, everybody accepted the scientific method as the best too=l that we ha=ve, [(H) And] was kinda letting the scientific method be the -- MICHAEL: [Mhm]. JIM: be the leader

Michael and Jim were discussing the role of scientists and the scientific method in Western societies. The arguments are quite complex and involve multifaced interpretations of modern technologies and their use. Michael's contribution ‘Mhm’ did not add any semantic content to Jim's argument or show any commitment to what Jim was saying. Instead, it had a simple positive commentary function: Michael indicated that he understood what Jim was saying. That is, he indicated that he believed that the representations both of Jim's language and of Jim's message were the same as Jim's. By comparing what he predicted and what Jim said, Michael was confident enough to acknowledge alignment over the concept SCIENTIFIC-METHOD. Using Michael's commentary, Jim in turn meta-represented that he was aligned with Michael. He, therefore, continued with his argument (as there was no need to reformulate). Note that Jim did not necessarily assume that they had the same opinion about the validity of his argument—the commentary related to understanding rather than opinions. If Michael instead provided a negative commentary, such as ‘The scientific what?’, then Jim would presumably have revised his plans, and perhaps clarified what he meant by ‘scientific method’.

Now consider a different example [18]:

3.
D: Wul did'e ever get married 'r anything?
C. Hu:h?
D: Did jee ever get married?
C: I have // no idea.

C did not hear the details of what D said and was not able to compare what she expected to what D uttered. Therefore they were not aligned linguistically. For example, if C failed to hear ‘married’, then C would have realized her representations of the phonology, syntax and semantics of that word were missing (and thus did not correspond to D's representations), and C would also have realized that her situation model did not correspond to D's situation model. Therefore, C meta-represented misalignment and uttered the negative commentary ‘Hu:h?’. In this case, the lack of linguistic alignment led to situation model misalignment which C meta-represented. In other words, not hearing the question properly made it impossible for C to map the question to events and people in the world. On hearing ‘Hu:h?’, D also meta-represented misalignment and reformulated her plans by repeating the question ‘Did jee ever get married?’. By uttering ‘I have // no idea’, C signalled that she believed she and D were aligned with respect to the questions, even if she had no answer.

## 5. Speakers distribute their work

Commentaries help the speakers share the effort of dialogue. Such distribution of labour minimizes the collaborative effort in dialogue—that is, the work that the speakers do together to reach communicative success [13]. As we have seen, speakers are under time pressure and might have only a vague idea of what their interlocutor can deal with in terms of complexity or background knowledge. Thus, speakers cannot plan the perfect utterance in advance. In dialogue, utterances are negotiated through feedback and eventually repaired, to fit the needs of the speakers involved.

Schegloff *et al*. [18] argued that speakers prefer to repair their utterances when they are suboptimal for themselves and their interlocutors and to initiate the repair before the interlocutors do. This means that speakers try to reduce the overall work of the dyad, by correcting themselves as soon as they detect an error. They can do so because they have access to what they are producing before the others do. In fact, speakers do not only predict and monitor their interlocutor's utterances but they also predict and monitor their own utterances. This means that they check if what they predicted they would say turns out to be what they actually utter. If it does not, they can reformulate their plan and correct themselves, typically before their interlocutor can correct them, using self-initiated repair [19]. Thus, the self-initiated repair is likely to reduce the collaborative effort needed to align on the goal of the conversation, as other-initiated repair requires more turns (and presumably words) to detect and correct the error.

When self-initiated repair is not possible, and speakers need their interlocutor to guide them, interlocutors often signal the source of trouble. The interlocutor who signals misalignment usually chooses a linguistic form that efficiently pinpoints where the problem might be. For example, when asking to disambiguate a personal pronoun such as ‘he’, the initiator might prefer a more specific repair such as the direct question ‘who?’ over less specific feedback, such as ‘huh?’ [23,24]. In addition, the location of the commentary provides evidence about the nature of the misalignment—for example, ‘huh’ is likely to indicate a problem with the immediately preceding contribution.

In sum, meta-representing alignment on individual contributions helps speakers highlight what components of the dialogue need to be reformulated or reframed to achieve situation model alignment. Moreover, it allows constant monitoring and distribution of speakers' efforts. Since alignment describes a relationship of similarity between mental representations of oneself and one's interlocutor, meta-representing alignment can be seen as a process that requires the combination of metacognition (i.e. with respect to oneself) and social cognition (i.e. with respect to one's interlocutor). Indeed, speakers do not only act on their representations by comparing what they planned to say with what they actually say (via metacognition, that leads, in case of discrepancy, to self-repair or disfluencies), but they also compare what they planned that their interlocutor would say and what this interlocutor actually happens to say (via social cognition, that leads, in case of discrepancy, to negative commentaries and subsequent reformulations, expansions or clarifications).

Contributions to dialogue often involve trial and error but can eventually lead to a balance between what is required of the two speakers. In other words, collaborative effort is minimized because it is distributed efficiently across the contributors. Notice that even when the conversation is asymmetrical, as when one of the two contributors is telling a story, the control of the dialogue remains appropriately distributed. Bavelas *et al*. [15] showed how storytellers benefit from their listeners' feedback, arguing for their role as co-narrators. Narrators told their story less well when distracted listeners provided suboptimal feedback. Feedback might be used to divide extended turns (such as anecdotes) into appropriate units that make it easier to signal and detect possible sources of misunderstanding [13,23]. Therefore, listeners directly control narrators’ turns by segmenting them and turning them into collaborative contributions. On the other hand, when narrators expect their story to be complex to understand, they proceed by installments—they segment their own contribution, predicting a positive commentary (an indication of understanding) or a negative commentary (an indication of misunderstanding) from the listener for each segment. Consider the following example [23]:

4. B. how how was the wedding-
A. oh it was it was really good it, was uh it was a lovely day
B. yes
A. and . it was a super place, . to have it . of course
B. yes -
A. And we went and sat in an orchard, at Grantchester, and had a huge tea *afterwards (laughs -)*
B. *(laughs --)*.
A. **uh**
B. **it does** sound, very nice indeed

By segmenting extended contributions, A made sure that she left space for B to indicate misalignment (which in turn led to no interruptions and/or complex rephrasing). At the same time, B kept indicating alignment, which allowed A to continue with her anecdote. In other words, A's utterances—and presumably her timing—left B with the possibility of commenting, which B in fact did. Thus, A did not reformulate her plans and instead told the story as she had prepared it.

## 6. Speakers negotiate abstract concepts

Speakers do not only meta-represent alignment to monitor the flow of the dialogue and adjust their representations but they also use meta-representations to negotiate and co-construct conceptual representations, an ability that is particularly relevant for abstract concepts.

Very different kinds of concepts are usually labelled as abstract (e.g. philosophical ones such as FREEDOM, emotional ones such as HAPPINESS, mental ones such as THOUGHT, mathematical ones such as MULTIPLICATION). However, they have important similarities: they cannot be easily pinned down to an external referent, their representation is often detached from sensory experience, and they tend to be affected by personal experiences, culture and contexts of use. In this sense, abstract concepts are arbitrary [6]: they are characterized by individual and cultural level variability and they cannot be easily represented by attending directly to entities in the world.

While traditional theories treat abstract and concrete concepts as categorically different [25–27], more recent embodied and distributional approaches propose that the relation between abstractness and concreteness is continuous and multidimensional, or even remove the distinction (for an extensive review see [4]). On the one hand, embodied theories claim that abstract concepts—in contrast with concrete ones—are more grounded in linguistic, social and metacognitive experiences [28–32]. On the other hand, distributional theories argue that the meaning of any concept is determined by linguistic co-occurrences. For example, the concept DOG is defined by co-occurring concepts such as WALK, BARK and FURRY, in the same way that the word DEMOCRACY is defined by co-occurring concepts such as POLITICS, STATE and REPRESENTATION.

What matters for our purposes is that the close relationship between abstract concepts and language use is a constant across different accounts. In fact, language also works as a glue that helps categorize everyday disparate experiences under the same concept [28], for example describing both being able to make political statements without fear of imprisonment and not having to work at a boring job in order to pay the bills as FREEDOM. Furthermore, given the variability, complexity and context dependency of abstract concepts, thinkers may need to rely on others in order to process them properly [30–32]. Since their referent cannot be easily found in the external world, their representation depends on what speakers agree when discussing them with other people.

In line with some theories of metacognition [30,32,33] which posit a monitoring and a regulative component in processing abstract concepts, we predict that the awareness of knowledge gaps (more common when abstract concepts are processed) can stimulate the comparison of representations (monitoring) and elicit (regulating) linguistic actions (i.e. commentaries), that can eventually ground the representation of such concepts. Additionally, we argue that the perception of misalignment triggers monitoring, which leads to negotiation between perspectives. More precisely, speakers make assertions and produce commentaries based on their judgement about the question ‘Am I aligned with you*’.* When the judgement is positive, it leads to positive commentary, a similar judgement by the addressee, and the dialogue moves on; when the judgement is negative, it leads to negative commentary, a similar judgement by the addressee, and (typically) an attempt to produce alignment. Iterations of assertions and commentaries enable speakers to come to shared conceptualizations. This process occurs for both concrete and abstract concepts, but is most extensive for abstract concepts, because of the prevalence of knowledge gaps and contestability. Such dyadic exchanges lead to the formation of abstract shared concepts that extend to groups and larger communities of diverse individuals, a phenomenon that can explain society-level mechanisms for which independent groups come to share similar category systems for complex phenomena [30,34,35].

It is possible to think about the tangram task—a dyadic reference game where one speaker is the director and the other is the matcher—as a process in which speakers agree on descriptions of figures whose name is contestable. Therefore there is a sense in which descriptions of such objects are abstract (even though such abstractness is rather different from the abstractness of DEMOCRACY or FREEDOM). In fact, such tangrams do not have conventional names, and speakers figure out together descriptions that are adequate for referring uniquely to each of them. Indeed, the game consists in naming and matching figures by agreeing on their salient features, for the sake of the task. In Clark & Wilkes-Gibbs [13], participants described the same set of figures six times. Let us consider the figure that is often referred to as ‘the ice skater’ (figure 2).

**Figure 2.**
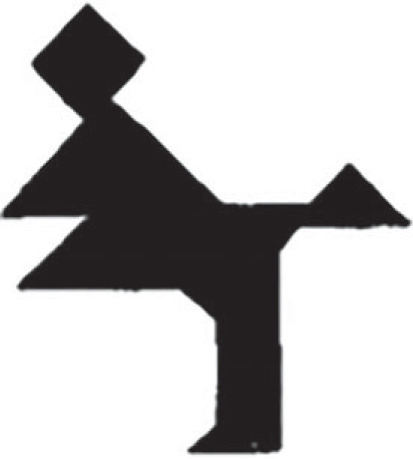
The ‘ice skater’ tangram.

Directors started by describing the figure, using long and detailed sentences (e.g. ‘the next one looks like a person who's ice skating, except they're sticking two arms out in front’). In subsequent turns, descriptions became simpler and simpler (e.g. ‘the fourth one is the person ice skating, with two arms'), until the interlocutors converged on a common description (the ice skater) and kept using it until the end of the game. The director and the matcher aligned on the representation ICE-SKATER by meta-representing alignment and using commentaries. In other words, when the matcher indicated that she meta-represented alignment by using a positive commentary, the director made use of this commentary to propose a simplification of the previous description. By contrast, when the matcher indicated that she meta-represented misalignment, the director reconceptualized his description, and typically planned the use of a different perspective that might have been easier for the director to align on. Thus, through various cycles of alignment and meta-representations, speakers built a shared and arbitrary representation of the figure. In other words, by negotiating and mutually shaping their representations, they conceptualized it as ‘the ice skater’.

We argue that the dynamics underlying the ice skater example as described here may give some insights into what speakers do when they have to deal with abstract concepts. Abstract concepts—such as politicized issues—are often not framed as given and they are open to contestability and collective building of meaning [31]. Consider, for example, the recurrent debates around the concept DEMOCRACY [36]. In a similar sense, with tangram figures, participants knew that different descriptions were available and did not just agree on their representations but built them dynamically. By signalling alignment on the label ‘ice-skater’—through linguistic or non-linguistic feedback—speakers recognized that the representation they had was similar enough to the one of their interlocutors and communicated a meta-cognitive state (we have the same understanding). This led to the perspective acceptance, which resulted in the routinization of the linguistic label ‘the ice skater’.

Now consider this last example from a debate between the sociologist Slavoj Žižek and the psychologist Jordan Peterson [37].

5.
Jordan Peterson: Well, I don't, I don't, first of all there's something you said five minutes ago or so, I think you were still at the podium that I agree with profoundly which is that happiness is a side effect it's notit's not a thing in itself it's something that comes upon you it's like an act of grace in some sense and senses.
Slavoj Žižek: I accept even the theological undertone of what you said.
Jordan Peterson: Ok, ok, ok,
Slavoj Žižek: No, no, the category of grace can be used in a perfect atheist sense.
Jordan Peterson: Yes.
Slavoj Žižek: It is one of the deepest categories.
Jordan Peterson: Yeah, well,
Slavoj Žižek: I'm sorry.
Jordan Peterson: Okay good, well, I would think that we could find agreement about that because partly because of your psychoanalytic background you know perfectly well that we're subject to forces within us that aren't of our voluntary control, and happiness is one of them.

While Peterson was trying to define HAPPINESS as ‘an act of grace’, Žižek meta-represented that they were aligned on what Peterson was saying, but did not necessarily share his stance. However, it is clear that the need to meta-represent alignment through commentaries drove the conversation off the path (moving the focus from what HAPPINESS is to how GRACE can be defined). At the same time, Peterson repeatedly provided commentaries on what Žižek uttered. These comments allowed both the speakers to represent alignment and to come back to the main argument. The context (a debate), the difference of background knowledge (given their respective disciplines, cultures and political perspectives) and the intrinsic abstractness and theoretical implications of the concepts of GRACE and HAPPINESS may be the reasons why the speakers felt the need to display whether they were aligned or not at nearly every assertion of the exchange so that they could manage to converge on conceptualizations that are similar enough to understand each other.

Our model of dialogue can provide a deeper understanding of the relationship between language, sociality, metacognition and alignment as mechanisms that are particularly relevant for the processing of abstract concepts. While representing a concrete concept such as LAMP in similar ways might just require both the speakers to see a lamp or to remember an exemplar that they have seen, touched, or used in the past, representing an abstract concept such as FREEDOM in similar ways requires the collaboration and negotiation that only dialogue allows. By interacting, speakers have the possibility to communicate metacognitive states and build shared representations that—while not necessarily implying consensus—support alignment.

## 7. Conclusion

Speakers understand each other because they have access to their own as well as to their interlocutor's representation of the dialogue they are involved in. By constant monitoring and shared control over each turn, speakers converge on similar representations of the world. Dialogue consists of iterated cycles of alignment and misalignment, which speakers systematically meta-represent through commentaries. Commentaries thus become prompts that speakers use to drive subsequent behaviour and to adjust their plans to each other's needs. After they meta-represent alignment, speakers can add new contributions as they planned and start new cycles; when they meta-represent failure of alignment, speakers reformulate their plans and correct their contributions to keep the dialogue on track. By commenting on each other's contributions, speakers also construct shared representations that arise from the interaction. We argued that such mechanisms are particularly relevant for processing abstract concepts and that our model can help explain their successful use.

## Data Availability

This article has no additional data.

## Footnotes

1 ^1^Here are some notation conventions used in the examples reported (in order of appearance). In extract 1 “:” shows that the speaker has stretched the preceding sound, “-” shows a sharp cut-off, “=” means that there is no gap between turns, and underlining indicates emphasis. In extract 2 “(H)” signals inhalation, “--” signals a truncated intonation unit and “[]” indicates overlapping speech. In extract 3, “//” indicates the point at which overlapping speech starts. In extract 4, “.” indicates a brief pause, “()” indicates contextual comments, “*” indicates paired instances of simultaneous talk, while more than one “-” stand for a combination of pauses. Extract 5 was manually transcribed by the authors.

2 Behaviour matching is not necessarily caused by automatic alignment and is sometimes used strategically to make the conversation smoother or easier to follow for interlocutors [10,11], but we will not focus on such cases here.

3 In the current and following sessions, we use the term *alignment* to refer to what we defined as *situation model alignment*, unless otherwise specified.
